# Application of a flipped classroom teaching model based on micro-videos in the standardized training of dermatological residents in China

**DOI:** 10.3389/fmed.2023.1250168

**Published:** 2023-10-13

**Authors:** Yang Li, Xian-fa Tang, Hui Cheng

**Affiliations:** ^1^Department of Dermatology, First Affiliated Hospital, Anhui Medical University, Hefei, Anhui, China; ^2^Key Laboratory of Dermatology, Anhui Medical University, Ministry of Education, Hefei, Anhui, China

**Keywords:** flipped classroom, standardized training, dermatological residents, micro-videos, theoretical knowledge, clinical practice skills

## Abstract

**Objective:**

To explore the effects of a micro-video-based flipped classroom teaching model on the standardized training of dermatological residents in China.

**Methods:**

A total of 78 residents who had received standardized training at the Department of Dermatology of the First Affiliated Hospital of Anhui Medical University were selected and randomly divided into an experimental group (39 residents) and a control group (39 residents). The experimental group received micro-video-based flipped classroom teaching, whereas the control group received traditional lecture-based classroom teaching. Scores relating to theoretical knowledge of dermatology, clinical practice skills, and the results of a questionnaire survey were used to evaluate the teaching effects.

**Results:**

The average score of the experimental group in the theoretical knowledge test (88.56 ± 5.80) was significantly higher than that of the control group (81.90 ± 7.45). Similarly, the average score of the experimental group in the clinical practice skills test (85.44 ± 5.97) was also significantly higher than that of the control group (78.46 ± 5.94). The results of the questionnaire survey showed that the experimental group exhibited significant improvements in learning interest, mastery of teaching content, communication skills, expression skills, clinical practice skills, autonomous learning, clinical thinking, clinical application, and team cooperation.

**Conclusion:**

Flipped classroom teaching based on micro-videos helped to improve the teaching effects of theoretical knowledge, clinical practice skills, and residents’ comprehensive ability during dermatological residents’ standardized training.

## 1. Introduction

The development of the Chinese economy and improvements in the standard of living has resulted in an increasing demand for qualified and higher-level dermatologists. Providing dermatologist training has been difficult owing to the diverse and complex types of dermatology (1).

Therefore, it is necessary to implement a standardized training system for residents to cultivate clinical physicians with solid medical theoretical knowledge and clinical practice skills as well as the ability to engage in autonomous learning, clinical thinking, and clinical application; thus, they would be well equipped to independently and normatively engage in the diagnosis and treatment of common diseases in their profession (2). The traditional standardized training process for residents is mainly based on a teacher-centered model that helps teachers to conduct lectures; however, it should be noted that training residents generally have low enthusiasm for autonomous learning and insufficient independent clinical diagnosis and treatment abilities (3). To improve their clinical thinking, diagnostic, and treatment abilities, it is thus necessary to explore new teaching models.

Micro-videos are short online videos with a maximum length of 20 min, which help explain relevant knowledge points (key points, difficulties, and doubts) (4). Before class, training residents can watch micro-videos through online platforms to learn relevant knowledge points that they can consult with their teachers in class. The concept of the flipped classroom is based on a student-centered teaching model that transfers knowledge before class and turns the classroom into a place for interaction and exchange of knowledge between teachers and students. The rationale behind the flipped classroom methodology is to increase student engagement with content, increase and improve faculty contact time with students, and enhance learning (5, 6). A new teaching model of flipped classrooms based on micro-videos has been applied to health professional education (7–9). The current study was designed to examine the effect of the flipped classroom teaching model, based on the micro-videos used during the standardized training of dermatological residents in China; we performed a randomized controlled study to investigate whether this new teaching model could improve the teaching effects related to theoretical knowledge, clinical practice skills, and residents’ comprehensive ability.

## 2. Materials and methods

### 2.1. Study subjects

A total of 78 residents who had received standardized training at the Department of Dermatology in the First Affiliated Hospital of Anhui Medical University were selected and randomly divided into an experimental group (*n* = 39) and a traditional didactic lecture control group (*n* = 39). The experimental group received micro-video-based flipped classroom teaching, whereas the control group received traditional lecture-based classroom teaching. Basic information of participants’ characteristics, including their sex, age, and dermatology entrance examination scores (Table 1), was obtained. There were no significant differences in sex, age, and dermatology entrance examination scores between the two groups (*p* > 0.05). All participants provided written informed consent. The study was approved by the Institutional Ethical Committee of the First Affiliated Hospital of Anhui Medical University (Approval number: PJ2022-10-55).

**Table 1:** Sample summary information in the study.

Characteristics	Experimental group	Control group	Statistics	*P*
Sample size	39	39		
Average age (years, mean ± s.d.)	25.21 ± 1.17	25.44 ± 1.10	*t* = −0.90	0.37
Sex, male/female	18/21	17/22	*x*^2^ = 0.05	0.82
Dermatology entrance examination scores (mean ± s.d.)	80.74 ± 9.01	81.54 ± 8.48	*t* = −0.40	0.69

### 2.2. Teaching methodology

The experimental and control groups were handled by the same teacher, and the teaching content of the two groups were the same.

#### 2.2.1. Design of the flipped classroom teaching model based on micro-videos

A flipped classroom teaching model based on micro-videos was applied to the experimental group. The micro-videos were created according to teaching material recommended by the National Health Commission for the standardized training of dermatological residents. A total of 24 theoretical knowledge and practical skill points were converted into micro-videos (Table 2). The micro-videos produced for developing clinical practice skills included information on clinical laboratory skills and treatment-operating skills. The content presented on clinical laboratory skills was as follows: (1) The content of dermatopathology slide review includes the pathological characteristics of eczema/dermatitis, psoriasis, erythema multiforme, lupus erythematosus, dermatomyositis, scleroderma, bullous dermatoses, and cutaneous vasculitis; (2) Fungal microscopic examination includes the microscopical characteristics of tinea capitis, tinea corporis, onychomycosis, pityriasis versicolor, and Malassezia folliculitis; and (3) Allergen test includes the operation and result interpretation of patch and puncture tests. In addition, the content presented on treatment-operating skills was as follows: (1) Skin biopsy includes the selection method and biopsy operating skills of the skin lesions; (2) Dressing change and hydropathic compress include adherence to sterile operating principles and mastery of operating procedures; and (3) Fluid nitrogen cryopreservation and carbon dioxide laser include mastery of the selection methods and treatment techniques for various types of warts and benign skin growth.

**Table 2:** Micro-video teaching contents in the study.

Chapter	Micro-video teaching contents	Video length
Theoretical knowledge of dermatology		
1	Viral dermatoses	10 min
2	Bacterial dermatoses	10 min
3	Fungal dermatoses	10 min
4	Eczema and Dermatitis	10 min
5	Urticaria	5 min
6	Drug Eruption	10 min
7	Papulosquamous Diseases	
	Psoriasis	8 min
	Erythema Multiforme	5 min
8	Autoimmune Diseases	
	Lupus erythematosus	10 min
	Dermatomyositis	6 min
	Scleroderma	5 min
9	Bullous Dermatoses	
	Pemphigus	5 min
	Bullous Pemphigoid	5 min
10	Cutaneous Vasculitis	
	Erythema Nodosum	4 min
	Anaphylactoid purpura	5 min
	Cutaneous small vessel vasculitis	5 min
11	Acne	7 min
12	Vitiligo	8 min
Clinical practice skills		
13	Clinical laboratory skills	
	Dermatopathology slide review	12 min
	Fungal microscopic examination	5 min
	Allergen test	5 min
14	Treatment-operating skills	
	Skin biopsy	6 min
	Dressing change and hydropathic compress	8 min
	Fluid nitrogen cryopreservation and carbon dioxide laser	10 min

##### 2.2.1.1. Before class

The teacher uploaded the micro-videos on WeChat, a free online information interaction platform developed by Tencent, a Chinese multinational technology and entertainment conglomerate and holding company. The videos were uploaded 14 days prior to when the face-to-face session was conducted. The training residents could watch the micro-videos online or download them to start learning before attending the class. They could leave messages on the WeChat platform about the video’s knowledge points that they found puzzling, which the teacher addressed in class.

##### 2.2.1.2. In class: application of the flipped classroom teaching model

First, the teacher divided the training residents into small groups and divided the questions raised on WeChat between the groups for discussion. The teacher asked the training residents to address these puzzling contents and guided them to understand the contents correctly.

Second, the teacher asked several questions, and conducted typical clinical case analyses and clinical practice skills operations based on the learning contents of the pre-class micro-videos. Each training resident group discussed the questions, clinical cases, and practice skills proposed by the teacher. The teacher guided the training residents to understand the concepts through classroom interactions, such as classroom discussions, communication, and answering questions.

Finally, the teacher set up an objective, written, classroom test to evaluate their degree of knowledge, which the training residents had to complete independently. In the formative multiple choice (objective) test used in this study, four options were provided for each question, and the training residents were required to select the correct answer. We randomly selected 10 questions from the standardized training exam question bank for this chapter, which are also applicable to the traditional didactic-lecture-based classroom teaching model, with a total obtainable score of 100 points.

#### 2.2.2. Design of the traditional didactic-lecture-based classroom teaching model

The traditional lecture-based classroom teaching model was implemented in the control group. The training residents were asked to preview the learning contents using the teaching notes provided before class. The teacher used traditional lecture-based classroom teaching, which also included clinical case analyses and clinical practice skills operations. The teacher further set up a classroom test, which was the same as the test undertaken by the experimental group.

### 2.3. Teaching effect evaluation

After the completion of all the teaching content, we conducted the theoretical knowledge examination and clinical practice skills test.

#### 2.3.1. Theoretical knowledge test

Theoretical knowledge examinations were conducted using closed-book written tests. We randomly selected examination questions from the standardized training exam question bank for dermatological residents in China. The test questions for the experimental and control groups were the same. The theoretical knowledge test form included multiple-choice questions, a fill-in-the-blanks test, essay questions, and case analysis questions, with a total obtainable score of 100 points. The contents of the test encompassed the diagnosis and treatment of common skin diseases to test the students’ theoretical knowledge and application analysis ability. Finally, the average test scores of the experimental and control groups were compared.

#### 2.3.2. Clinical practice skills test

Clinical practice skills included clinical laboratory and treatment-operating skills. The total obtainable score in the skill evaluation test was 100 points. Clinical practice skills assessments were evaluated by two associate chief physicians in our dermatology department. The evaluation indicators were quantified to reduce evaluation error.

#### 2.3.3. Questionnaire survey

After all the teaching contents were completed, we distributed the questionnaire to both the study groups and asked them to fill out the form in person (i.e., paper-based).

### 2.4. Statistical analyses

#### 2.4.1. Sample size estimation

To determine the sample size of this study, we conducted a preliminary experiment. Based on the results of the preliminary experiment, we detected an average score difference of 8.1 between the experimental and control groups, with a standard deviation of 6.51, using a two-tailed t-test of difference between means with 90% power, and a significance level *α* = 0.05. Furthermore, an experimental samples-to-control ratio of 1:1 was used. The sample size was calculated using the formula below. As a result, we determined that at least 14 experimental samples and 14 control samples would be required. We decided to utilize 39 experimental samples and 39 control samples.


$$n={\left|\frac{\left(Z_{\alpha /2}+Z_{\beta }\right)\sigma }{\delta }\right|}^{2}\;\left(Q_{1}^{-1}+Q_{2}^{-1}\right)\;\alpha =0.05,\beta =0.1$$

#### 2.4.2. Questionnaire reliability evaluation

The internal consistency reliability of the questionnaire was estimated using Cronbach’s alpha coefficient, using SPSS version 14.0. The Cronbach’s α coefficients of the experimental and control group questionnaires were 0.979 and 0.983, respectively.

#### 2.4.3. Statistical analyses of test and the questionnaire survey

Statistical analyses were performed using SPSS version 14.0. Test scores are presented as means ± standard deviation (s.d.). The scores of the two groups are compared using an independent-sample *t*-test. The questionnaire survey data are presented as sample numbers (n, %) using the chi-square test. Statistical significance was set at *p* < 0.05.

## 3. Results

### 3.1. Results of the theoretical knowledge test and clinical practice skills test

As shown in Table 3, the average score of the experimental group in the theoretical knowledge test (88.56 ± 5.80) was significantly higher than that of the control group (81.90 ± 7.45) (*p* < 0.001). Similarly, the average score of the experimental group in the clinical practice skills test (85.44 ± 5.97) was also significantly higher than that of the control group (78.46 ± 5.94) (*p* < 0.001).

**Table 3:** Results of theoretical knowledge test and clinical practice skills in the study.

Characteristics	Theoretical knowledge	Clinical practice skills
Average score of experimental group (mean ± s.d.)	88.56 ± 5.80	85.44 ± 5.97
Average score of control group (mean ± s.d.)	81.90 ± 7.45	78.46 ± 5.94
t	4.41	5.17
*P*	<0.001	<0.001

### 3.2. Results of the questionnaire survey

A total of 78 questionnaires were distributed, with a response rate of 100%. The questionnaire results are presented in Table 4. The results of the questionnaire survey show that the experimental group had significant improvements in learning interest, the mastery of teaching content, communication skills, expression skills, clinical practice skills, autonomous learning, clinical thinking, clinical application, and team cooperation.

**Table 4:** Results of the questionnaire survey in the study.

Statement number	Statement	Experimental group (*n* = 39)			Control group (*n* = 39)			*x* ^2^	*P*
		Agree (%)	Uncertainty (%)	Disagree (%)	Agree (%)	Uncertainty (%)	Disagree (%)		
Q1	Stimulate your learning interest	32 (82.05%)	5 (12.82%)	2 (5.13%)	18 (46.15%)	13 (33.33%)	8 (20.51%)	11.08	0.004
Q2	Enhance your autonomous learning ability	33 (84.61%)	4 (10.26%)	2 (5.13%)	17 (43.59%)	12 (30.77%)	10 (25.64%)	14.45	<0.001
Q3	Improve your mastery of teaching contents	30 (76.92%)	5 (12.82%)	4 (10.26%)	18 (46.15%)	12 (30.77%)	9 (23.08%)	7.81	0.02
Q4	Improve your abilities of clinical thinking and clinical application	32 (82.05%)	4 (10.26%)	3 (7.69%)	16 (41.03%)	13 (33.33%)	10 (25.64%)	13.87	<0.001
Q5	Develop your communication and expression skills	34 (87.18%)	3 (7.69%)	2 (5.13%)	15 (38.46%)	8 (20.51%)	16 (41.03%)	20.53	<0.001
Q6	Enhance the team cooperation ability	35 (89.74%)	2 (5.13%)	2 (5.13%)	13 (33.33%)	9 (23.08%)	17 (43.59%)	26.38	<0.001

These results reveal that residents prefer micro-video-based flipped classroom teaching to the traditional lecture-based classroom teaching.

## 4. Discussion

The standardized training of resident physicians is a specialized training to deepen medical graduates’ theoretical knowledge and clinical practice skills within their area of specialization prior to becoming qualified doctors (3). At present, most clinical teachers at hospitals still employ the traditional didactic-lecture-based classroom teaching style that focuses on one-sided “indoctrination.” Training residents passively accept the information presented, and the method is not conducive to improving residents’ knowledge and skills (3). To improve teaching effects, it is necessary to explore new teaching models.

In this study, we explored the effects of the micro-video-based flipped classroom teaching model on the standardized training of dermatological residents in China. The effectiveness of this teaching model was evaluated through the tests conducted to assess residents’ theoretical knowledge and clinical practice skills and the questionnaire survey; the results were compared with those obtained for the traditional teaching model. The results of the theoretical knowledge and clinical practice skills tests indicated that the learning outcomes of residents within a flipped classroom based on micro-videos was significantly better than that of residents within a traditional teaching setting (*p* < 0.001). Furthermore, the results of the teaching feedback questionnaire indicated that the experimental group had a significant increase in their interest in learning, mastery of teaching content, communication skills, expression skills, clinical practice skills, autonomous learning, clinical thinking, clinical application, and team cooperation (*p* < 0.05). Consistent with our findings, previous studies have shown that the teaching outcomes resulting from a flipped classroom model based on micro-videos was significantly better than those of the traditional didactic-lecture-based classroom for the education of health professionals in fields such as pharmacology (10), emergency medicine (11), neuroscience (12), and anesthesiology (13).

The reason for the flipped classroom teaching effect being superior to the traditional teaching effect could be attributed to multiple factors. First, vivid micro-videos can present boring and complex medical content in a vivid form, stimulating students’ interest in learning and enthusiasm for autonomous learning (10, 14). Second, it gives the students the opportunity to make full use of their time in the classroom by taking part in applied activities and learning, and thus, gain better comprehensive ability. After watching the micro-videos prior to attending class, training residents enter the classroom with questions that they are eager to answer, thereby encouraging them to actively participate in classroom activities (15). In class, the teacher assigns the collected students’ questions to each student group for discussion. Meanwhile, the teacher asks several questions based on the learning contents of the pre-class micro-videos. Each group discusses the questions proposed by the teacher. The teacher provides timely guidance and answers questions, ultimately achieving problem solving, which not only helps stimulate the students’ thinking (16) and enhance their ability to analyze and solve problems (17), but also helps to deepen their understanding and mastery of clinical knowledge (18). Simultaneously, through interaction, cooperation, and exchange among students, as well as between students and teachers, students’ communication and team cooperation abilities are cultivated (15). Third, a flipped classroom could effectively solve problems arising from significant differences in the theoretical level and practice abilities among the students. The use of the traditional lecture-based classroom teaching model by teachers attempts to seek a “one size fits all” solution, which is difficult to achieve when trying to meet the needs of students with different learning abilities; especially those with lower levels of ability, who cannot keep up with the rest of the class. Hence, flipped classrooms can effectively solve this problem, whereby prior to attending class, students can practice self-learning using micro-videos at their own pace. If the contents are unclear or complex, they can repeatedly watch and consult relevant materials until they fully understand them before moving on to the next section. In this way, the students’ level of classroom learning is roughly the same, reducing the level of learning difference among students. For questions that arise during self-study, group discussions among students and communication between students and the teacher, can help to ultimately solve the problem, and achieve the effect of “teaching according to one’s aptitude” (19).

The current study has a number of limitations. The evaluation of the flipped classroom model is partially performed via the participating residents’ self-assessment, such as communication skills. Therefore, an updated evaluation system, which includes measures of core competencies other than knowledge acquisition, will be necessary to better evaluate the effectiveness of the flipped classroom model (9).

## 5. Conclusion

In conclusion, the flipped classroom teaching model based on micro-videos, used during the standardized training of dermatological residents, helped to improve the teaching effects related to residents’ theoretical knowledge, clinical practice skills, and comprehensive ability. Hence, further improvements to the application of the teaching model can be made in future.

## Data availability statement

The original contributions presented in the study are included in the article/supplementary material, further inquiries can be directed to the corresponding author.

## Ethics statement

The studies involving humans were approved by the Institutional Ethical Committee of the First Affiliated Hospital of Anhui Medical University. The studies were conducted in accordance with the local legislation and institutional requirements. The participants provided their written informed consent to participate in this study.

## Author contributions

YL conceived this study, conducted micro-video-based flipped classroom teaching and traditional lecture-based classroom teaching, and performed the statistical analysis. YL and X-fT participated in the design and management. YL and HC enrolled the participants. All authors contributed to the article and approved the submitted version.
